# Effect of cool vs. warm dialysate on toxin removal: rationale and study design

**DOI:** 10.1186/s12882-015-0017-5

**Published:** 2015-02-27

**Authors:** Vaibhav Maheshwari, Titus Lau, Lakshminarayanan Samavedham, Gade P Rangaiah

**Affiliations:** National University of Singapore, 21 Lower Kent Ridge Road, Singapore, 119077 Singapore; Division of Nephrology, Department of Medicine, National University Health System, 1E Kent Ridge Road, Singapore, 119228 Singapore; Department of Chemical and Biomolecular Engineering, National University of Singapore, 21 Lower Kent Ridge Road, Singapore, 119077 Singapore; Current Affiliation: Renal Research Institute, 315 East 62nd Street 4th Floor, New York, NY 10065 USA

**Keywords:** Hemodialysis, Dialysate temperature, Cool/Warm dialysate, Inter-compartmental resistance, Spent dialysate, Toxin removal

## Abstract

**Background:**

Cool dialysate is often recommended for prevention of intra-dialytic hypotensive episodes in maintenance hemodialysis (HD) patients. However, its effect on toxin removal is not studied. It is known that inter-compartmental resistance is the main barrier for toxin removal. Cool dialysate can potentially increase this resistance by vasoconstriction and thus impair the toxin removal. The aim of this trial is to compare the toxin removal outcome associated with cool vs. warm dialysate.

**Method/design:**

This study is based on the hypothesis that dialysate temperature, a potential maneuver to maintain hemodynamic stability during HD, may influence inter-compartmental resistance and hence, toxin removal. Only stable HD patients will be recruited for this study. The quantum of removed toxins will be assessed by the total spent dialysate, which is a gold standard to quantify the efficacy of a single dialysis session. Collected samples will be analyzed for urea, creatinine, phosphate, β_2_-microglobulin, and uric acid. The study is a single center, self-controlled, randomized prospective clinical research where 20 study subjects will undergo 2 dialysis sessions: (a) cool dialysis with dialysate at 35.5°C, and (b) warm dialysis with dialysate at 37°C. Pre- and post-dialysis blood samples will be collected to quantify the dialysis adequacy and toxin reduction ratio.

**Discussion:**

This is the first clinical research to investigate the effect of dialysate temperature on removal of both small and large-sized toxins. Successful completion of this research will provide important knowledge pertaining to dialysate temperature prescription. Results can also lead to the hypothesis that cool dialysate may help in by preventing intra-dialytic hypotensive episodes, but prolonged prescription of cool dialysate may lead to comorbidities associated with excess toxin accumulation. The new knowledge will encourage for personalized dialysate temperature profiling.

**Trial registration:**

Clinicaltrials.gov Identifier - NCT02064153.

## Background

Improving toxin removal can potentially improve the hemodialysis (HD) patient outcome. In this context, the decades old HD procedure progressed from low efficiency low-flux dialysis to high efficiency high-flux dialysis and currently towards increased acceptance for convection based hemodiafiltration (HDF). However, in all these extracorporeal renal replacement therapies, toxin removal is primarily impaired by inter-compartmental resistance [1,2]. Overcoming this resistance seems to be the single most effective method for improved toxin removal. Intra-dialytic exercise may reduce this resistance by vasodilation. Exercise increases the cardiac output and reduces peripheral vascular resistance as the vasculature dilates. This vasodilation may be augmented by increased body core temperature due to exercise [3]. However, intra-dialytic exercise is still considered an intervention in routine dialysis setting, not a norm. Also, HD patients with significant muscle wasting may not be able to exercise during dialysis. How then can we induce the vasodilation without exercise or can we increase the body core temperature without exercise?

Dialysate temperature is an easy maneuver which can change the blood temperature, a surrogate of body core temperature. Warm dialysate can increase the body core temperature, resulting in vasodilation and increased mobilization of sequestered toxins to intravascular compartment. The contrary physiological change i.e. vasoconstriction can similarly be induced by cool dialysate and this is often recommended for prevention of intra-dialytic hypotensive (IDH) episodes. IDH is defined as a fall in systolic blood pressure below 90 mmHg or a drop of more than 20 mmHg that results in clinical symptoms, and occurs in 20-30% of treatments [4-6]. Cool dialysate induced vasoconstriction may reduce the toxin mobilization from remote inaccessible body compartments to intravascular compartment, thus hindering the toxin removal, which is contrary to the fundamental objective of HD. Hence, although cool dialysate helps in prevention of intra-dialytic episodes in short-term, prolonged usage may lead to poor patient outcome by impaired toxin removal. If cool dialysate does hinder toxin removal, then for at least 70-80% non-hypotensive HD patients, the benefits of warm dialysate may be realized.

Few studies have investigated the effect of cool dialysate on urea removal, and found that urea based dialysis adequacy is largely unaffected by dialysate temperature [7,8]. Nevertheless, it is suggested that urea based adequacy marker is not a true representative of toxins removal [9,10], as urea is too small in size and experiences negligible inter-compartmental resistance. Urea kinetics can also be explained by perfusion term or by regional blood flow alone [11,12], i.e. change in inter-compartmental resistance will have little effect on its mobilization. The same does not apply for large-sized toxins [13]. Even other small-sized toxins do not conform to the kinetic behavior shown by urea [14,15]. Interestingly, a clinical trial pertaining to removal of both small and large sized toxins via dialysate temperature manipulation has never been performed until now. Hence, the aim of this clinical study is to compare the toxin removal outcome for cool vs. warm dialysate for both small and large-sized toxins.

## Methods and design

### Study design and settings

The study is a single center, self-controlled, randomized study involving patients undergoing conventional high-flux dialysis. Patients are not informed about the dialysate temperature *a priori*; however, some subjects may report the warm or cool sensation based on their experience. The study will be conducted at satellite dialysis center of the National University Hospital (NUH), Singapore.

### Ethics approval and quality assurance

The domain specific review board affiliated with the National Healthcare Group (NHG), Singapore has approved the trial. The study will undergo routine quality assurance review conducted by the ethics board. The ethics board will also receive timely progress status report from the principal investigator and will be promptly informed of any adverse events owing to the study intervention. The grantor will also receive timely report of study progress.

### Patient recruitment

The warm dialysate may result in IDH episodes; hence, only stable HD subjects with no prior history of IDH will be recruited for the proposed study. The dialysis nurses will provide the list of all stable patients in the past one month. The principal investigator will review the database of these stable patients. The patients with history of angina, heart-attack, or chronic obstructive pulmonary diseases (COPD) will not be recruited for the trial. Though stable on HD, these patients may be unstable during warm dialysis session. The residual renal function of study subjects should be negligible (defined by urine output of less than 200 mL/day). Only those subjects, who follow 3 times per week dialysis schedule, will be recruited. Subjects satisfying our mentioned criteria will be contacted. The study administrator will explain about the intervention and the potential benefits as well as harms. Agreed subjects will sign the patient information sheet and consent form. A copy of the consent form will be provided to the subject. Total 20 subjects will be recruited for the study.

### Inclusion criteria

1. Adult patients male or female (Age > 21 years, Age < 70 years)
2. Minimum dialysis vintage of 3 months
3. Stable on hemodialysis
4. Blood access capable of delivering the blood flow rate greater than 250 mL/min

### Exclusion criteria

1. History of recurring or persistent hypotension in the past 1 month
2. Pregnant woman
3. Severely hypertensive patients (SBP > 180 mmHg and/or DBP > 115 mmHg)
4. Severely hypotensive patients (SBP < 100 mm Hg and/or DBP < 60 mmHg)
5. Paradoxically hypertensive patients whose BP increases by more than 20% of baseline during dialysis (in the past 1 month)
6. History of recent myocardial infarction or unstable angina (within the past 6 months)
7. Significant valvular disease, i.e. severe aortic stenosis and moderate-severe mitral regurgitation
8. Patients with end stage organ disease e.g. COPD, recent or debilitating CVA
9. Patients with Left Ventricular dysfunction, Chronic Heart Failure and older age group more than 70 years
10. Patient with recent stroke (within the past 6 months)
11. History of known arrhythmia
12. Participation in another clinical intervention trial
13. Unable to consent

Dialysis hypotension may occur in one of three clinical patterns: (i) acute (episodic) hypotension defined as a sudden drop of systolic blood pressure below 90 mmHg or of at least 20 mmHg with accompanying clinical symptoms, (ii) recurrent – as detailed above but prevailing in a minimum 50% of dialysis sessions, and (iii) chronic, persistent hypotension in which interdialytic systolic blood pressure is maintained at less than 90–100 mmHg.

### Study interventions and randomization

All recruited subjects will undergo two study sessions: (i) cool dialysis with dialysate temperature at 35.5°C and (ii) warm dialysis with dialysate temperature at 37°C. The dialysis duration for all sessions will be 240 min. In total, 40 study sessions will be conducted. The maximum and minimum interval between two sessions respectively is one month and one week. Minimum of one week gap is maintained to avoid any carry over effect from the previous study session. All dialysis sessions will be performed using high flux (single-use) dialyzer and the Fresenius 4008S machine. Except dialysate temperature, the dialysis prescription and patient medications (phosphate binder, medicine for hypertension, erythropoietin, etc.) will remain unchanged. Patients will also be advised to keep their dietary intake fairly constant during the study period.

Patients are generally fluid overloaded on the beginning of the week i.e. Monday/Tuesday and target ultrafiltration (UF) will generally be set higher on those days. This higher fluid removal requirement may sometimes result in cramps or hypotensive episodes. Warm dialysate may aggravate this situation by vasodilation; hence, no study session will be performed on Monday/Tuesday. The study sessions for a particular patient will be performed on the same day of the week. All the study sessions will be conducted in randomized order, but patient’s existing dialysis schedule will not be disturbed for the purpose of the study.

### Data collection

#### Blood and dialysate sampling

During each study session, pre- and post-dialysis blood samples will be collected. These samples will be analyzed for both small and large-sized uremic toxins, namely, urea, creatinine, phosphate, β_2_-microglobulin, and uric acid. The dialysate flow will be kept at 500 mL/min. Total spent dialysate will be collected in a large container. To assess the quantum of removed toxin mass in a particular study session, the spent dialysate will be analyzed for the mentioned toxins. The spent dialysate collection is the gold standard to ascertain session adequacy. All the samples will be sent to NUH Laboratory Medicine Department for analysis. The pre- and post-dialysis blood samples will be used for calculating the toxin reduction ratio and urea based dialysis adequacy.

### Treatment modification

Study sessions will be discontinued, should adverse-effects such as acute hypotensive episode (defined by sudden change in blood pressure – drop of 20 mm Hg or SBP < 100) or chest pain, uneasiness, or cramps manifest. Although these are anticipated to be at low event occurrence rate, the event will be medically recorded and dealt with usual clinical practice. During the session, if a patient complains about temperature being too high or too low, then the dialysate temperature will be adjusted to his/her routine prescription and subject will be removed from the study. A report of all such adverse events will be provided to the ethics board.

### Outcome measure

#### Primary outcome measure

The objective of this clinical study is to investigate the effect of dialysate temperature on removal of both small and large-sized toxins. To quantify the removed toxin mass in an individual study session, the spent dialysate sample will be analyzed for toxin concentration. The removed toxin mass will potentially depend on the pre-dialysis toxin concentration. If pre-dialysis toxin concentration is high then there is a possibility that more toxin mass will be removed; hence removed toxin mass assessed by spent dialysate will be scaled with respect to pre-dialysis serum toxin concentration. This scaled removed toxin mass for both study sessions will be compared using standard statistical *t*-test. The toxin reduction ratio will also be calculated using pre- and post-dialysis toxin concentrations.

#### Secondary outcome measure

**Figure 1:**
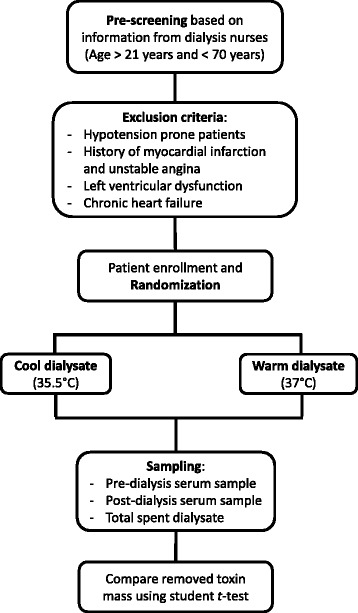
**Schematic flow diagram of the clinical study.**

Dialysate temperature can potentially change the hemodynamic response. For each study session, patient blood pressure (BP) and heart rate (HR) will be recorded at every 5 minutes during the whole study session. If any sudden change in BP or HR is noticed, the dialysis nurses will be informed and appropriate clinical action will be taken. If hemodynamic response is indicative of hypotensive episode, then study session will be suspended. The schematic flow chart of the study is presented in Figure 1.

## Discussion

Cool dialysate is often recommended as a potential maneuver for controlling and/or preventing the incidence of intra-dialytic hypotensive (IDH) episodes. Cool dialysate reduces the body core temperature which leads to vasoconstriction and consequently helps in maintaining hemodynamic stability during dialysis treatment [5]. It is suggested that IDH occurs when cardiovascular response cannot compensate for large volume losses due to excessive UF rate. This can occur under the circumstances when UF rate exceeds the plasma refilling rate and persists for long enough such that reduction in blood volume reaches the critical threshold. Importantly, this critical threshold differs for an individual patient and is influenced by individual cardiovascular response [16]. This emphasizes the need of individualized dialysate temperature for prevention of IDH incidence. Before one aim for individualized dialysate temperature profile, it is important to study the effect of vasoconstriction on toxin removal.

It has been mentioned that blood cooling has adverse effect on solute disequilibrium [12]. In a study of unstable HD patients, cool dialysate (35°C) was associated with significantly greater increase in peripheral vascular resistance [17]. This increased vasoconstriction can potentially reduce the mobilization of toxins from remote compartments to intravascular compartment, thus reducing the dialysis efficacy. Cool dialysate may be good for 4 hour dialysis, but, in the long term, it may culminate into various complications due to less efficient removal of uremic toxins. One such co-morbidity is amyloidosis due to prolonged accumulation of middle molecule marker, β_2_-microglobulin. It will not be wrong to say that cool dialysate focuses only on one aspect of the dialysis treatment i.e. prevention of IDH, but it must be weighed against the plausible unwanted negative influence of impairing an equally important aspect of treatment i.e. uremic toxin removal. Effect of cool dialysate on urea clearance has been studied, and its removal was not significantly affected by cool dialysate [4,7,18,19]. It is because urea is too small in size to experience significant inter-compartmental resistance from cellular membrane and/or capillary endothelium. Also, the rapid equilibration of urea across the cell membrane is essentially due to facilitated transport by selective urea transporters [20]; such transporters do not exist for other similar sized toxins. Hence, it is imperative to study the effect of dialysate temperature on toxin removal other than urea. Interestingly, there is no clinical study which has investigated the effect of dialysate temperature on toxin removal. The proposed clinical trial intends to fill the gap by comparing the toxin removal outcome for cool and warm dialysate. Contrary to cool dialysate induced vasoconstriction, warm dialysate will result in vasodilation and open capillary surface. This will contribute towards enhanced toxin mobilization from remote inaccessible compartments to intravascular compartment and their subsequent removal in the dialyzer. One should also note that warm dialysate may result in incidence of IDH, and so only stable on HD subjects will be recruited for the trial.

Selection of marker toxin is an important aspect in assessing dialysis efficiency. As mentioned earlier, since urea clearance is unaffected by vasoconstriction or by cool dialysate, a better marker should be chosen. In this trial, we are considering both small and large sized uremic toxins, namely, urea, creatinine, phosphate, β_2_-microlgobulin, and uric acid. Last but not least, the method to assess dialysis efficiency is important to compare the two study sessions. Number of criterion like toxin reduction ratio, dialysis adequacy based on Kt/V_urea_, and post-dialytic rebound can be used. The gold standard is to collect the whole dialysate or fraction of it continuously [21], analyze a sample from whole spent dialysate and quantify the removed toxin mass. In this trial, we will collect the whole dialysate in large drums.

Successful completion of this project will encourage clinicians to refrain from one-size-fit-all approach of dialysate temperature. Selby and McIntyre have also recommended the prospect of individualized dialysate temperature [22]. Further research may lead to patient-specific dialysate temperature profiling, which will not only prevent the incidence of intra-dialytic hypotensive episodes but adjusted to also maximize the toxin removal.
